# Supplementary material for: Shade provision and its influence on water intake and drinking behaviour of Nellore cattle in feedlot in a tropical environment
Source: PLoS One. 2025 Sep 12;20(9):e0331238. doi: 10.1371/journal.pone.0331238 (PMC12431413; doi:10.1371/journal.pone.0331238)
Supplement: S2 File — (DOCX) [file pone.0331238.s002.docx]

| **Reviewer’s comments** | **Authors’ response** |
| --- | --- |
| The authors describe drinking behaviour of beef cattle with/without shade availability, a topic that has not been well researched despite close links with welfare and, therefore, is of high importance considering rising temperatures due to global warming. The article is in general well written. | Thank you for your valuable comments and time dedicated to review our manuscript. We have addressed all your comments. A clean version of the revised version highlights the changes in blue text. |
| However, there is a lack of consistency in wording that needs to be addressed and authors provide only n=2 per group which is a very small sample size. In many cases it is unclear if values are calculated per pen or per animal, this must be more transparent. | Values are calculated per individual animal. Text has been added to clarify the RFID tags were uses to track individual cattle. |
| It is described that in heat animals start to play with water instead of drinking – how did you ensure to measure intake or consumption? | Individual water intake was measured using the Intergado™ System (Intergado Ltd., Contagem, Minas Gerais, Brazil), which employs RFID technology to track individual cattle and water flow meters installed in the troughs to precisely record the volume of water consumed during each drinking event. The system logs only the water volume that flows through the trough during a drinking event, as detected by the flow meters, ensuring that non-consumptive interactions, such as splashing or playing with water, are not recorded as intake. Additionally, the water troughs were designed and tailored to beef cattle drinking behaviour, minimizing spillage and supporting accurate measurement water intake. |
| For me, the dataset is this small, that I suggest changing the original research article into a short communication. | The journal does not specifically take short communications, though it can occasionally be a prefix in the title and may be down to editor discretion here. Given that smaller sample sizes are more prone to Type II errors, the extent to which significant differences and associations have been found in our study is, we believe, relatively convincing, especially given the little research done in this area. We would also note that the sample size is higher than numerous other livestock behaviour full papers in this journal:  N= 20 ([10.1371/journal.pone.0285933](https://doi.org/10.1371/journal.pone.0285933))  N = 19 ([10.1371/journal.pone.0131632](https://doi.org/10.1371/journal.pone.0131632))  N = 15 ([10.1371/journal.pone.0265037](https://doi.org/10.1371/journal.pone.0265037))  N = 24 (10.1371/journal.pone.0118617)  N = 18 (10.1371/journal.pone.0144583) |
| L17 Please move “steers” before brackets : Nellow Steers (Bos indicus) | Done. |
| L19 rephrase to “allocated in two groups” | Done. |
| L 26 Hard to read, please shorten or split in two sentences: Environmental factors such as Air temperature, humidity, and solar radiation influenced water intake in both groups, with higher air temperatures increasing water intake due to greater drinking frequency and higher relative humidity reducing water intake by decreasing visit frequency. | Rephrased and split. |
| L29 water demand, not requirements – please pay attention to wording consistency throughout the manuscript | The expression “water demand” was used consistently throughout the document. |
| L38 please also indicate the threat of heat stress for animal health and welfare | Added. |
| L42 space lacks at the end of the sentence before bracktes with source | Space added. |
| L46 Cattle use evaporative cooling to dissipate heat load. However, evaporative cooling increases the cattle´s need for water to maintain homeostasis [6], consequently increasing the demand of the production systems. | Rephrased. |
| L59 please delete water before drinking behavior and in the following text. Generally maintain the same phrase (drinking behavior OR water drinking behavior, better drinking behavior; water intake OR water consumption, better water consumption) | “drinking behaviour” used throughout the manuscript.  We prefer to keep “water intake” consistently throughout the document since this is how the response variable (WI) was defined and presented in tables and graphs. |
| L83 months, not mo. | Changed. |
| L88 May not an unequal number of animals per group per see affect outcomes as drinking frequencies, behaviors etc ? Did you check your data regarding a potential effect of access per animal? | Group sizes were balanced except for one pen, which had one fewer animal due to an illness after the adaptation period, with no replacement available. While we acknowledge the theoretical possibility that group size differences could affect access to resources and influence drinking behaviours, this was not observed in our study. The water troughs, designed and sized by Intergado Ltd. according to manufacturer specifications for beef cattle, provided adequate capacity and access for all animals in each pen, minimising competition. The Intergado™ System recorded consistent metrics across groups. Statistical analysis of these variables showed no significant differences attributable to the minor variation in group size, confirming that access to the troughs was not restricted and that drinking frequencies and behaviours remained unaffected. |
| L98 Please provide, if possible, any information of the wells´ water quality | There is no information available. |
| L103 water consumed instead of drunk, please change through the whole document | “drunk” replaced throughout the manuscript. |
| How was water consumption measures? Which devices were used, how were the water trough sized? | In the original manuscript, we briefly mentioned the use of the Intergado™ System (Intergado Ltd., Contagem, Minas Gerais, Brazil) for monitoring individual water intake, as referenced in Chizzotti et al. (2015).  The Intergado™ System utilises RFID technology to identify individual animals and water flow meters attached to the troughs to measure the volume of water consumed during each drinking event. The troughs were designed and sized by Intergado Ltd. to accommodate the cattle’s drinking behaviour, ensuring sufficient access and capacity for accurate measurement, though specific dimensions were not detailed in our study as they followed the manufacturer’s standard specifications for beef cattle. We have included this new information in the manuscript. Similarly, we enhanced the description of the feed intake masurement. |
| L119 (“W) | Amended. |
| Fig 1: Provide the size of pens in the figure, it would additionally be desirable to have a picture/sizes of the trough/shade constructions. | As mentioned in the manuscript, each pen is 400 m^2^. We have added photos of all the relevant features in the pen. |
| L129 Was normal distribution of variables checked by wilcox rank test? Please specify. Please indicate the packages used, also to illustrate the figures | The Wilcox rank test does not require a normal distribution and thus testing is not necessary.  The decision to use non-parametric tests for this was a factor of both sample size and non-normal distributions of variables and the desire for consistency in testing methodologies. Text has been added in this regard.  We have also changed the model used for the data to a zero inflated model to better account for the distribution of the data.  Citations for R packages added. |
| L141 ranged from.. to.. Please use protected spaces before units | Amended |
| L162 was is meant by V= ? | This is Wilcoxon’s V, a test statistic. It represents the sum of the ranks of positive differences between the treatment groups. |
| L162 who is “them”? Individuals of this group? Generally, use (cattle OR animals OR individuals and maintain consistency) | Amended |
| L164 What is meant by spending 39% more?time? Here also use protected spaces before ALL units in the whole document. | Sentence slightly amended. The expression “spending xx% more time” means the duration (e.g., in minutes or seconds) was greater by that percentage compared to the other group. |
| LL170 p = 0.053 = 0.05 = significant, no tendency needed here. | We respectfully disagree with the reviewer. The limit for significance is 0.05. Only values up to 0.050 are considered significant. Hence, given that 0.053 > 0.050, this is a trend only. |
| L174 X and Y correlated positively (r = , p < ) | Changed. |
| L176 delete “than with shade” | Done |
| L177 what means 5-6 fold? | The term "5-6 fold" means an increase of 5 to 6 times the original value (i.e., a multiplication factor of 5 to 6). This phrase is a common scientific shorthand. |
| L185-193 This information is doubled, as its shown in Fig. 3. However, I understand why mentioning key correlations. You may indicate exact r values at the end of the phrases to provide additional information and/or remove the Fig. in Supplementary File. | Here we are describing the results in terms of correlations (as we do with tables). We agree that the exact r-value should be provided and this has now been added for the significant correlations highlight However, we prefer to maintain the figure in the main document as this provides additional information. A supplementary table with the exact r and p values has been added. |
| L200 specify “affected” | Changed to ‘associated’ |
| 224 highlight, however, that | We disagree with the suggestion. The phrase is correct in grammar and meaning. |
| 242 consistent wording- better demand than requirement | Done throughout the whole manuscript. |
| 243 please delete/rephrase “elsewhere” | Done. |
| 252 replace it with shade | Done |
| L256 Rephrase “water search frequency” the frequency cattle seek for water? | Rephrased to enhance clarity, but the expression “water search frequency” was retained as it’s grammatically correct. |
| L310 Please indicate a source for this statement | Added. |
| 395-397 repetition of “justify investments” | Sentences rephrased. |
