# Supplementary material for: Shade provision and its influence on water intake and drinking behaviour of Nellore cattle in feedlot in a tropical environment
Source: PLoS One. 2025 Sep 12;20(9):e0331238. doi: 10.1371/journal.pone.0331238 (PMC12431413; doi:10.1371/journal.pone.0331238)
Supplement: Attachment — Submitted filename: Revision note PLOS One round 2.docx [file pone.0331238.s004.docx]

| **Reviewer#2’s comments** | **Authors’ response** |
| --- | --- |
| Reviewer #2: L84: Use body weight (BW) instead of liveweight (LW) for consistency and clarity. | Body weight (BW) has been used throughout the whole document. |
| L91–94: Why was the initial BW estimated using a regression based on only two time points? This approach is typically used for calculating average daily gain (ADG) when three or more data points are available. Alternatively, consider using the average BW from two consecutive weighing days. | In our study, animals were weighed at 21-day intervals from the acclimatisation period to minimise handling stress, which precluded additional weighing on day 1 of the assessment period. To estimate initial BW, we assumed a linear growth rate between the two consecutive weighing days bracketing the assessment period (i.e., the weighing days before and after day 1). A regression based on these two points enabled us to estimate BW specifically for day 1. Conversely, averaging the BW from these two weighing days would yield a midpoint estimate, which would not accurately reflect the BW on day 1, as it is not equidistant between the two weighing dates. We believe this regression approach provides a more precise estimate of initial BW for our analysis. |
| L96: Were animals fed ad libitum or were they restricted to a specific amount? Please clarify this in the text. | Animals were fed ad libitum, with daily monitoring of feed refusals in the trough to minimise feed wastage. Clarification added in Line 94. |
| L98–100: What was the nutrient composition of the diet? Include a table with detailed dietary composition. | The nutritional composition has been added to the main text (L113-118) |
| L104–106: Please rephrase this sentence for clarity and flow. | Sentence rephrased (L102-104) |
| L123–126 (Fig. 1): Why were the +S pens all located on the same side (east) of the facility? Was there no randomization in pen assignment? | Each of the four groups was randomly allocated to one of the four pens. This has now ben clarified in the text (L87-88). |
| L136–137: I strongly disagree with the approach taken here. While individual animal data can be collected for dry matter intake (DMI) and water intake (WI), the pen should be considered the experimental unit when comparing treatments. The shade treatment was applied at the pen level, not at the individual animal level, and thus treatment effects cannot be independently assessed at the animal level. | We acknowledge the challenge of treating individual animals as experimental units when the shade treatment was applied at the pen level. In animal science research, resource constraints often limit full replication at all levels, making it common to treat individual animals within pens as experimental units for measures such as dry matter intake and water intake, which can be accurately recorded at the individual level. This approach is consistent with several studies published in Plos ONE (e.g., DOI: 10.1371/journal.pone.0308182; 10.1371/journal.pone.0305671; 10.1371/journal.pone.0200165). Furthermore, Reviewer 3’s positive evaluation of the study’s robust design and appropriate statistical analyses suggests that our approach is methodologically sound within the context of the field’s conventions. To address the reviewer’s concern and enhance transparency, we have revised the Discussion section (L409-413) to explicitly highlight the issue of pseudoreplication and to urge caution in interpreting the results, ensuring readers are aware of the limitations of this approach. We believe these revisions address the concern while maintaining the study’s scientific integrity, as supported by Reviewer 3’s assessment. |

| **Reviewer#3’s comments** | **Authors’ response** |
| --- | --- |
| Reviewer #3: I recommend rejection of this manuscript due to a lack of innovation. Although the study was **well designed and the statistical analyses are appropriate**, the manuscript does not offer any new or relevant insight into the topic under investigation. The discussion is well grounded in the literature, showing that what they have discovered can easily be found in the literature. But overall, I don't believe the work has the level of impact required for publication in this journal.  As the authors themselves recognize, future studies could explore the long-term effects of shade provision on cattle performance using a more integrated approach. For example, incorporating landscape-level shade mapping (based on sun position), GPS tracking of cattle movements, water intake, weather conditions and animal health and performance metrics would result in a more comprehensive and impactful study. I encourage authors to consider expanding the scope of their research in this direction to strengthen future submissions. | We thank the reviewer for their constructive feedback and for acknowledging the robust design, appropriate statistical analyses, and well-grounded discussion in our manuscript. We also appreciate their suggestions for future research directions.  Regarding the manuscript’s lack of innovation, we respectfully disagree and believe the study makes a valuable contribution to the literature. As noted by Reviewer 1 in a previous round of revisions, “*The authors describe drinking behaviour of beef cattle with/without shade availability, a topic that has not been well researched despite close links with welfare and, therefore, is of high importance considering rising temperatures due to global warming*.” Our study addresses this under-researched area by evaluating the effects of artificial shading on confined beef cattle under tropical conditions, incorporating individual monitoring of water and feed intake as well as water-related behavioural assessments. As highlighted in our manuscript, while numerous studies on cattle shading exist, the majority focus on dairy cattle in temperate climates and often lack detailed individual-level data on intake and behaviour. In contrast, research on confined beef cattle in tropical environments, particularly in the context of a climate emergency where performance challenges are intensified, is scarce. Validating management practices such as artificial shading in these conditions holds substantial productive, economic, and social relevance, addressing critical welfare and sustainability concerns under rising global temperatures.  We believe the current study, as supported by Reviewer 1’s assessment of its novelty and relevance, provides a meaningful and timely contribution to the field and merits publication in this journal. We have highlighted this in the Discussion section (L423-426). |
