# Supplementary material for: Shade provision and its influence on water intake and drinking behaviour of Nellore cattle in feedlot in a tropical environment
Source: PLoS One. 2025 Sep 12;20(9):e0331238. doi: 10.1371/journal.pone.0331238 (PMC12431413; doi:10.1371/journal.pone.0331238)
Supplement: Attachment — Submitted filename: Reviewer response PLOS ONE drinking behaviour.docx [file pone.0331238.s005.docx]

**Reviewer #3:**

1: Should environmental variables be considered part of the Results or Methods section? In my opinion, the THI within the pen should be treated as a result, since it was affected by the treatment (shade). However, the other environmental variables (e.g., ambient temperature, rainfall, etc.) should remain in the Methods section, as they are not controlled by the experiment and are more appropriately described as background conditions.

Answer: The THI and the main environmental variables were obtained from a single met station at the research centre, as there were no temperature or humidity sensors inside the pen. Therefore, we believe this information should remain in the Methods section. In the Discussion section, we address some potential changes in environmental variables that can be expected under the shade.

2: I suggest presenting the diet ingredients and nutrient composition in a separate table rather than in the text.

Answer: Thank you for the suggestion. We have now created a Table for the diet description.

3: This is just a general comment for consideration in future studies, no changes to the current manuscript are needed because de explanation is already in the limitations. While I understand and agree that the individual tracking system (feed and water troughs) allowed for the measurement of individual-level responses, I respectfully disagree with the classification of the individual animal as the experimental unit. This issue led to a lengthy discussion "Variability in water intake and drinking behaviour"
From a statistical and conceptual standpoint, the experimental unit is defined as the smallest unit to which a treatment is applied. In this case, the shade treatment was pen.

Answer: Thank you for your insightful comment regarding the classification of the experimental unit in our study. We acknowledge and agree with your concern that, from a statistical and conceptual standpoint, the pen is the smallest unit to which the shade treatment was applied. However, in animal experimentation, practical constraints often necessitate compromises. In our study, we used two pens per treatment due to logistical and resource limitations, as replicating more pens would require additional animals and more expensive equipment, such as feed and water troughs. Increasing the number of pens by reducing the number of animals per pen could alter the social and environmental dynamics within the pen, potentially misrepresenting the conditions typical of a feedlot setting and raising questions about the applicability of our findings.

This design reflects a trade-off between statistical rigor, resource availability, representativeness of feedlot conditions, and adherence to the 3Rs (Replacement, Reduction, and Refinement) principles of ethical animal research. We appreciate your understanding, as noted in our limitations section, and will consider this feedback for refining experimental designs in future studies.
